# Identifying Return to Work Self-Efficacy Trajectories in Employees with Mental Health Problems

**DOI:** 10.1007/s10926-021-09979-2

**Published:** 2021-05-12

**Authors:** Lena Horn, Maitta Spronken, Evelien P. M. Brouwers, Renée S. M. de Reuver, Margot C. W. Joosen

**Affiliations:** 1 Tranzo Scientific Center for Care and Wellbeing, Tilburg School of Social and Behavioral Sciences, Tilburg University, Professor Cobbenhagenlaan 125, 5037 DB Tilburg, The Netherlands; 2 Faculty of Psychology, Open University of the Netherlands, Valkenburgerweg 177, 6419 AT Heerlen, The Netherlands; 3 Department Human Resource Studies, Tilburg School of Social and Behavioral Sciences, Tilburg University, Warandelaan 2, 5037 AB Tilburg, The Netherlands

**Keywords:** Return to work self-efficacy, Mental health problems, Sick leave, Trajectories, Latent class growth analysis

## Abstract

*Purpose* Return to work self-efficacy (RTW-SE) is a strong predictor of return to work (RTW) in employees with mental health problems (MHPs). However, little is known about the development of RTW-SE during the RTW process. In this study, we aimed to identify RTW-SE trajectories in the year following sick leave in employees with MHPs and provided a description of the trajectories in terms of personal and work characteristics, and RTW status. *Methods* This multi-wave study included 111 employees with MHPs. RTW-SE was measured at baseline, and at 3, 6, and 12 months follow-up with the RTW-SE scale for employees with MHPs. *Results* Latent class growth analysis revealed six trajectories. In three trajectories employees had increasing RTW-SE scores, namely (class 1) low start, moderate increase, (class 3) moderate start, small increase and (class 5) moderate start, steep increase. The other trajectories were defined by (class 2) persistently high, (class 6) persistently low, and (class 4) decreasing RTW-SE scores over time. Employees across the various trajectories differed significantly with respect to RTW status, and personal and work characteristics measured at baseline, including age, gender, and type of MHP. Less favorable trajectories (class 4 and 6) were characterized by higher age, a higher prevalence of anxiety disorder and lower RTW rates. The most favorable trajectory (class 2) was characterized by a higher proportion of stress-related disorders and less major depression diagnoses. *Conclusions* Large heterogeneity exists in terms of RTW-SE trajectories in employees with MHPs and significant differences were found across the trajectories regarding personal and work characteristics, and RTW status. Insights into RTW-SE trajectories and their attributes are important to advance more effective and personalized RTW treatment for employees with MHPs.

## Introduction

In many Western countries, mental health problems (MHPs) pose a major challenge among working populations [1]. Thirty percent of adults experience MHPs, such as depression, anxiety disorder and stress-related disorders at some point during their lifetime [2]. The negative impact on the individual, and economic costs for society and employers are high. The majority of these costs does not stem from health care use, but can be attributed to sickness absence, reduced work functioning and unemployment [2]. Therefore, it is highly important to improve our knowledge of factors that facilitate return to work (RTW) in this population. One factor that is highly predictive of RTW and plays a key role in the RTW process, is return to work self-efficacy (RTW-SE) [3, 4]. The present study aims at gaining more insight about individual variation of RTW-SE during the RTW process, as knowledge on this topic is still limited.

In brief, self-efficacy is the belief that an individual has in her/his capacity to successfully perform a certain behavior [5, 6]. In the context of RTW, employees must feel confident about their abilities to resume their work and perform their job successfully [4]. An advantage of RTW-SE, in contrast with other predictors of RTW such as age, gender and education is that it can be modified through evidence-based interventions [7, 8]. Earlier studies have emphasized the function of low RTW-SE as an obstacle to RTW in various populations, including groups with mood and anxiety disorders, burnout and job stress [9–16]. Thus, changing obstructive self-efficacy cognitions may play an important role in effective treatment plans and promoting RTW [4, 7, 15, 17].

Only a few studies have investigated the development of RTW-SE, during the RTW process in employees with MHPs [14, 16, 17]. In general, an overall linear increase of RTW-SE over a period of 3 to 18 months has been reported in these studies. However, as gradual work resumption and relapse are common, return to work itself does not necessarily show a simple linear increase [18, 19]. Examining more complex change patterns that may underlie such linear increases may therefore be valuable. Lagerveld and colleagues showed that both high baseline RTW-SE and RTW-SE increase are important predictors of faster full RTW [16]. Similarly, several intervention studies showed that apart from RTW-SE levels at the start of sick leave, an increase in RTW-SE due to an intervention, was equally predictive of faster work resumption [20–24]. Interventions consisted of elements of work-focused cognitive behavioral therapy, provided by a psychologist or occupational physician (OP).

As the majority of previous studies has been cross-sectional and did not investigate more complex patterns of RTW-SE over time, more comprehensive information about individual variability in RTW-SE may be important for health care providers to guide individual RTW more effectively. Employees may differ in their levels of RTW-SE to start with and RTW-SE levels may develop differently over time rather than follow a simple linear pattern [17]. Hence, more knowledge about different RTW-SE trajectories and what characterizes these trajectories may help to improve and personalize RTW treatment.

The aim of the present study is to increase the knowledge about return to work self-efficacy trajectories in employees with mental health problems. Therefore, we firstly examine which distinct RTW-SE trajectories can be identified among employees on sick leave due to MHPs in the first 12 months after sick leave. Secondly, we aim to provide a description of employees in the different trajectories, in terms of personal characteristics, work characteristics, and return to work status. Greater knowledge of different trajectories and their characteristics may contribute to the development of more effective, personalized RTW interventions.

## Methods

### Study Context

In the Dutch social security system, the employer compensates sickness absence for two years with at least 70% sickness absence compensation. During this period the employee cannot be dismissed. When employees report sick to their employer, employees are required to visit a certified occupational physician (OP), hired by the employer, for independent assessment. Within six weeks, the OP has to provide an analysis of the work (dis)ability problem and within eight weeks, the employer and employee formulate a reintegration plan, including work modifications and gradual return to work. During the reintegration phase, the employee has regular consultation meetings with the OP, who provides the employee and the employer with advice about work accommodations and measures to promote RTW.

### Study Design

This study involved secondary analyses of data that was part of a larger study, a clustered randomized controlled trial (RCT). That study investigated the effectiveness of guideline-based care by OPs in the recovery process in employees with MHPs (trial registration ISRCTN 86605310) [17]. A detailed description of the design and procedure of that study can be found elsewhere [17]. In short, occupational physicians took part in an interactive training that focused on overcoming barriers to adhere to the existing guideline “The management of mental health problems of workers by occupational physicians” [25]. Approval from the Medical Research Ethics Committee of St. Elisabeth Hospital in Tilburg (MREC number 1162) was obtained and all participants gave written informed consent. The research design of the current study incorporated longitudinal data from employees with MHPs in the year following sickness absence. Participants were followed from the start of sickness absence and received follow-up questionnaires measuring RTW-SE and several personal and work characteristics, and RTW status after 3, 6 and 12 months.

### Procedure

Eligible employees were selected from the sick leave registration system of the participating Occupational Health Service (OHS). All employees diagnosed with MHPs and aged between 18 and 64 were invited to participate in the study after their first consultation meeting with the OP. According to the guideline for OPs, the first consultation with the OP should preferably take place within two weeks and always within 6 weeks after the first day of sickness absence as determined in the Dutch Gatekeeper improvement act [26]. Furthermore, the average time between the first day of sickness absence and inclusion to the study was 8.2 weeks (*SD* = 3.41, *N* = 80). During a structured telephone interview, interested employees were screened based on several criteria. Employees came from a wide variety of companies in various sectors (e.g. health care, education, municipality, engineering, industry). Inclusion criteria were: (1) MHPs were the primary reason for sick leave diagnosed by an OP, (2) on current sick leave after the first meeting with OP and (3) adequate command of the Dutch language. Exclusion criteria were: (1) being suicidal and (2) a physical problem being the primary reason for sick leave. Employees who met these criteria received a baseline questionnaire shortly hereafter. To identify RTW-SE trajectories, data regarding RTW-SE at baseline (T0), after 3 (T1), 6 (T2) and 12 months (T3) were used. For personal and work characteristics, we used baseline measurements. RTW status was measured at baseline and at 3, 6, and 12 months.

### Measurements

Return to work self-efficacy was assessed with the RTW-SE scale for employees with MHPs, consisting of 11 items (*α* = 0.94) rated on a 6-point Likert scale ranging from 1 (*totally disagree*) to 6 (*totally agree*) [4]. The instructions asked the employees to imagine that they start working their full contract hours again tomorrow. Considering their present state of mind, they had to answer statements about their RTW expectations. A sample item is: “If I resumed my work fully tomorrow, I expect that I will be able to perform my tasks at work”. Employees that had already returned to work received the same questions to map their perception of self-efficacy about work.

### Personal Characteristics

Demographic characteristics included: age at baseline, gender, and education level (low = primary and secondary school, high = upper secondary school or higher education).

Previous diagnosis of a mental disorder was based on the following question “Have you previously been diagnosed with a mental health disorder? “ (1 = yes, 2 = no).

The PRIME-MD which allows the identification of 18 diagnostic categories in line with the Diagnostic and Statistical Manual of Mental Disorders (DSM-IV), was used as diagnostic assessment tool [25, 27]. In line with the evidence-based guidelines for Dutch occupational physicians, stress-related disorders were defined as having mental health symptoms, but not meeting the criteria of a mental disorder according to the DSM-IV [28]. Most patients in this category reported being burned-out or overworked. Minor mood disorders were defined as suffering from one of the following: recovering from major depression, minor mood disorder, and dysthymia. Anxiety disorders in our sample were either generalized anxiety disorder or anxiety disorder not otherwise specified. Somatoform disorders included multi-somatoform and somatoform disorders not otherwise specified.

Burnout symptoms were self-assessed with the Utrecht Burnout Scale (UBOS) [29]. The scale consists of 15 items rated on a 7-point Likert scale ranging from 0 (*never*) to 6 (*daily*). The scale was used as a continuous, unidimensional construct and internal consistency was high in our sample (*α* = 0.90).

Work ability was measured with a self-report single-item question of the Work Ability Index (WAI) [30].

Coping style was self-assessed with the shortened version of the Utrecht Coping List measuring active problem-focused coping, emotional coping, and distraction-oriented coping (UCL) [31]. The scale consists of 14 items measured on a 4-point Likert scale ranging from 1 (*hardly ever*) to 4 (*very often*). The active, problem-focused coping subscale consists of 5 items (*α* = 0.83), the emotional coping subscale consist of 5 items (*α* = 0.74), and the distraction-oriented coping scale consists of 4 items (*α* = 0.75).

Additionally, as the data comes from a clustered RCT, experimental group was included as a characteristic (0 = control group, 1 = experimental group).

### Work Characteristics

Job content was assessed retrospectively with the Dutch version of the Job Content Questionnaire (JCQ), a self-report questionnaire that measures social and psychological characteristics of the job [32]. The scale consists of 30 items in 5 subscales and responses were measured on a 4-point Likert scale ranging from 1 (*totally disagree*) to 4 (*totally agree*). The psychological job demands scale consists of 5 items (*α* = 0.60). The physical job demands scale consists of 5 items (*α* = 0.84), the decision latitude scale consists of 9 items (*α* = 0.73), the social support scale consists of 8 items (*α* = 0.81), and the job insecurity scale consists of 3 items (*α* = 0.79).

### RTW Status

RTW status was self-assessed at four time points and further categorized into three variables: no RTW (0% of the contracted working hours), partial RTW (1–99%) and full RTW (100%). Number of working hours was included as number of contracted working hours per week.

### Statistical Analysis

Latent class growth analysis (LCGA) in Latent GOLD 5.1 was used to identify RTW-SE trajectories. In LCGA, persons are grouped together based on similar growth patterns that differ from the patterns of other groups [33]. RTW-SE was used as continuous dependent variable, time (in months) was used as predictor. As the sample included 95 cases with complete data (4 time points) and 16 cases with incomplete data (3 time points), missing values for the latter were estimated with posterior mode estimation.

Models with linear, quadratic, and cubic parameters with 1–7 classes were tested and analyses were run with 160 random start values and 250 iterations to overcome the problem of local solutions [34, 35]. Relative model fit was assessed with Bayesian Information Criterion (BIC), with lower values indicating better models. Bootstrap Likelihood Ratio Test (BLRT) was used to examine if the k-1-class models was rejected in favor of the k-class model. Entropy was evaluated to determine class separation, with higher values (> 0.6) indicating higher certainty of correct classification [33]. Last, class size (> 5%) and conceptual meaningfulness of the models were assessed [33, 36].

Based on their posterior membership probabilities, participants were assigned to one trajectory. To characterize the trajectories, associations between baseline variables and RTW status over time with the trajectories were examined. Three-step approach was used for continuous data and maximum likelihood (ML) for categorical data [37, 38]. The three-step approach corrects classification errors that are introduced from assigning individuals to latent classes by a two bias-adjustment procedure [38]. Wald tests were used to determine whether there were significant differences between the latent classes regarding these characteristics (*p* < 0.05).

## Results

### Personal and Work Characteristics

Table 1 presents descriptive statistics for personal and work characteristics. The average age of this sample (*N* = 111) was 46.8 years (*SD* = 10.8; Dutch population: 44.3*, SD* = 12.5) [39]*.* There were somewhat more women than men in our sample (36.9%; Dutch population: 44.8%). The majority of the employees had a high level of education (73.8%, Dutch population: 67.8%) and average contracted working hours of 32 h per week (*SD* = 7.3). Additionally, most employees were diagnosed with major depression. If employees had multiple diagnoses, they were rated multiple times in Table 1. Lastly, somewhat less than half of the sample had been previously diagnosed with an MHP.

**Table 1:** Descriptive statistics for personal and work characteristics at baseline (N = 111)

	*M* (*SD*)	Range
Age (years)	46.9 (10.8)	26–63
Working hours per week	32 (7.3)	8–42

	*N*	%
Gender (male)	41	36.9
Level of education		
Low	28	26.2
High	79	73.8
History of MHPs		
Yes	47	42.3
Type of MHPs (PRIME-MD)^a^		
Stress-related disorder	19	17.1
Major depression	62	55.9
Minor mood disorder	41	36.9
Anxiety disorder	47	42.3
Somatoform disorder	34	30.6
Experimental group (yes)	53	47.7

*M* mean, *SD* standard deviation; *N* sample size; ^a^Employees can be diagnosed with multiple MHPs

### Identifying Return to Work Self-Efficacy Trajectories

**Fig. 1:**
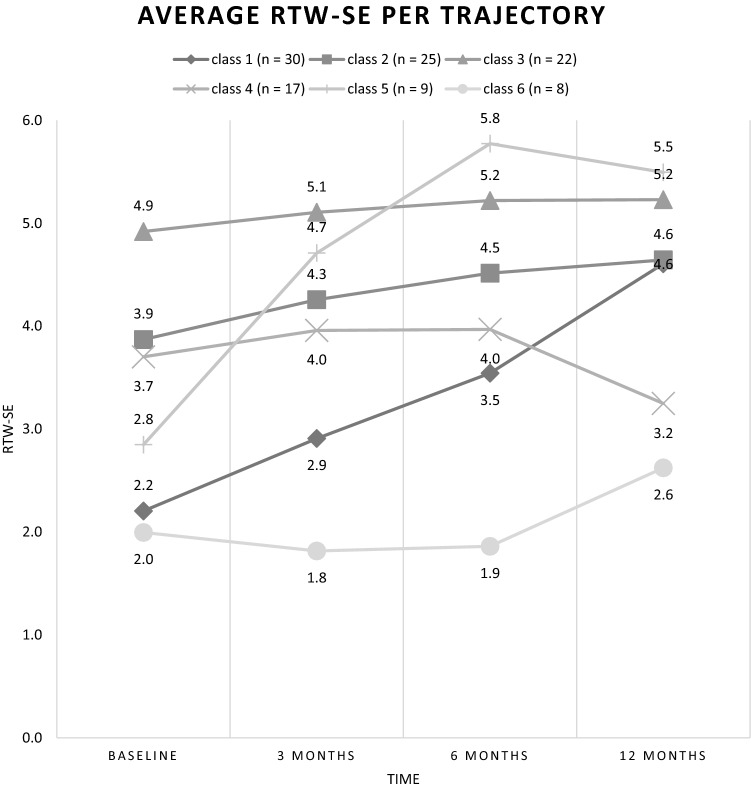
The average RTW-SE levels for each trajectory of the six-class model

The quadratic parameters outperformed the linear and the cubic parameters. BIC was lowest for the 6-class quadratic model and BLRT was significant (*p* < 0.05), confirming that the 6-class model fits the data significantly better than the 5-class model. Although the BLRT continued to improve, the addition of a seventh class served only to split one class into two parallel trajectories without substantive difference in RTW-SE levels. With an estimated proportion of classification errors of 0.08 and entropy *R*^2^ of 0.87, classification statistics were good and revealed the 6-class model as optimal (Table 2, Fig. 1).

**Table 2:** Fit statistics for one to seven class latent growth models

Model	No. of parameters	BLRT	BIC	Entropy *R*^2^	*Sample size per class (n)*
1-class	4	p < .001	1331,304	0,1147	111
2-class	9	p < .001	1208,776	0,5783	60/51
3-class	14	p < .001	1193,7121	0,6589	56/48//7
4-class	19	p < .001	1178,879	0,7124	54/25/24/8
5-class	24	p < .001	1165,634	0,7834	42/26/26/9/8
**6-class**	**29**	**p < .001**	**1159,347**	**0,8129**	**30/25/22/17/9/8**
7-class	34	p < .05	1162,446	0,8234	29/26/17/16/9/8/6

Sample sizes per class, based on most likely class membership

The selected model is in bold

*BLRT* Bootstrap Likelihood Ratio Test; *BIC* Bayesian Information Criterion

Figure 1 presents the six RTW-SE trajectories. Trajectory 1 (27% of the sample) showed low RTW-SE scores at baseline with a moderate increase over time (*low start, moderate increase*)*.* Trajectory 2 (23%) was characterized by persistently high RTW-SE scores from the start (*persistently high).* Trajectory 3 (20%) started at moderate levels, followed by a small increase over 12 months (*moderate start, small increase).* Trajectory 4 (15%) started at moderate levels, followed by a decrease between months 6 and 12 (*moderate start, decrease)*. Trajectory 5 (8%) was characterized by moderate start levels, followed by a steep increase (*moderate start, steep increase)*. Trajectory 6 (7%) had persistently low scores over time (*persistently low).*

### Characteristics of the Return to Work Self-Efficacy Trajectories

Significant differences between the 6 classes were found on age, gender, type of MHP, RTW, number of working hours, and physical job demands (all p ≤ 0.05). We found no significant differences between the trajectories for education, history of MHPs, coping, work ability, burnout scores, experimental group, and most domains of the job content questionnaire: psychological job demands, decision latitude, social support at work, and job insecurity. The most important differences are summarized below (see Table 3 for means, proportions per trajectory and significance test).

**Table 3:** Differences between the six RTW-SE trajectories and personal characteristics

Client characteristics	Sample (N = 111, 100%)	1. Low start, moderate increase (n = 30, 27%)	2. Persistently high (n = 25, 23%)	3. Moderate start, small increase (n = 22, 20%)	4. Moderate start, decrease (n = 17, 15%)	5. Moderate start, steep increase (n = 9, 8%)	6. Persistently low (n = 8, 7%)	Wald	p	Post-hoc
*Personal characteristics*										
Age (years), *M*	46.9	43.6	48.3	47.9	49.5	42.1	49.9	31.12	.021*	2, 3, 4,6 > 1, 5
Gender (male), *n* (%)	41 (36.9%)	12.5%	47.3%	22.2%	72.6%	31.9%	8.3%	19.34	.002**	2 > 1,6; 4 > 1, 3, 6
Education (high), *n* (%)	78 (73.6%)	81.7%	68.9%	68.4%	71.6%	89.1%	62.6%	2.87	.072	–
History of MHPs (yes), *n* (%)	47 (42.3%)	52.1%	22.3%	31.5%	69.4%	33.7%	50.8%	9.82	.080	–
Work Ability, *M*	3.9	4.3	4.0	3.4	4.5	3.1	2.9	4.04	.54	–
Active problem focused coping *M*	2.8	2.8	3.1	2.70	2.8	2.8	2.7	7.76	.17	–
Distractive Coping, *M*	2.1	2.1	2.2	2.1	2.1	2.3	2.3	2.00	.85	–
Emotional coping, *M*	2.2	2.1	2.2	2.2	2.1	2.2	2.5	2.23	.82	–
History of MHPs (yes), *n* (%)	47 (42.3%)	52.1%	22.3%	31.5%	69.4%	33.7%	50.8%	9.82	.080	–
Minor depression (yes), *n (%)*	35 (31.5%)	34.5%	32.2%	31.1%	11.5%	52.3%	37.9%	16.16	.006**	1, 2, 6, 5 > 4; 5 > 2, 3
Stress-related disorders, *n *(%)	19 (17.1%)	16.2%	53.5%	2.0%	17.1%	0.3%	0.2%	114.37	4.9e−23***	1,4 > 5, 6; 2 > 1, 3, 4, 5, 6
Major depression, *N *(%)	62 (56.8%)	59.4%	22.9%	66.0%	58.0%	82.3%	87.8%	12.96	.024**	2 < 1, 3, 4, 5, 6
Somatoform disorder (yes), n (%)	34 (30.6%)	26.5%	32.34%	6.4%	54.4%	37.3%	50.2%	81.72	2.3e−13***	1 > 3; 4,5,6 > 1, 2, 3
Anxiety disorder, *n *(%)	46 (41.4%)	34.2%	32.0%	41.0%	72.3%	39.5%	35.4%	27.16	5.3e−5***	4 > 1, 2, 3, 5, 6
Burnout (UBOS), *M*	2.9	3.0	3.0	3.1	3.0	3.0	2.6	1.75	.88	–

*p < 0.05; **p < 0.01; ***p < 0.001; *M* = mean, *n* = sample size; No differences between trajectory groups were found regarding experimental/control group of the original study

**Table 4:** Differences between the six RTW-SE trajectories and work characteristics

Client Characteristics	Sample (n = 111, 100%)	1. Low start, moderate increase (n = 30, 27%)	2. Persistently high (n = 25, 23%)	3. Moderate start, small increase (n = 22, 20%)	4. Moderate start, decrease (n = 17, 15%)	5. Moderate start, steep increase (n = 9, 8%)	6. Persistently low (n = 8, 7%)	Wald	p	Post-hoc
*Work characteristics*										
Work hours, *M*	31.9	30.9	36.0	29.5	32.4	29.9	30.8	17.29	.004**	2 > 1, 3
Physical job demands, *M*	1.6	1.9	1.6	1.6	1.4	1.9	1.4	16.30	.006**	1 > 4, 6; 5 > 4, 6
Psychological job demands, *M*	2.8	2.8	2.9	2.8	2.8	3.0	2.8	2.08	.84	–
Decision latitude, *M*	3.0	3.1	3.1	3.1	2.8	3.2	3.0	5.11	.40	–
Social support, *M*	2.8	2.7	3.0	2.7	2.9	2.6	2.8	9.18	.10	–
Job insecurity, *M*	1.6	1.6	1.6	1.6	1.6	1.8	2.1	5.22	.39	–

*p < 0.05; **p < 0.01; ***p < 0.001; *M* = mean, *n* = sample size

**Table 5:** Differences between the six RTW-SE trajectories and RTW status

Client Characteristics	Sample (n = 111, 100%)	1. Low start, moderate increase (n = 30, 27%)	2. Persistently high (n = 25, 23%)	3. Moderate start, small increase (n = 22, 20%)	4. Moderate start, decrease (n = 17, 15%)	5. Moderate start, steep increase (n = 9, 8%)	6. Persistently low (n = 8, 7%)	Wald	p	Post-hoc
*RTW status*										
Partial RTW T0, compared with no and full RTW, *n* (%)	46 (43.8%)	48.2%	53.2%	43.6%	29.2%	66.2%	14.7%	4.85	.43	–
Full RTW T0, compared with partial and no RTW, *n* (%)	16 (15.1%)	0.1%	30.7%	6.6%	23.9%	21.8%	13.3%	49.65	1.6e-9***	2, 3, 4, 5, 6 > 1
Partial RTW T1, compared with no and full RTW, *n* (%)	46 (43.8%)	55.9%	29.4%	59.9%	41.2%	33.7%	24.4%	5.70	.34	–
Full RTW T1, compared with partial and no RTW, *n* (%)	35 (33.3%)	19.6%	72.0%	29.1%	31.2%	41.2%	13.4%	10.02	.075	–
Partial RTW T2, compared with no and full RTW, *n* (%)	35 (33.0%)	38.8%	3.0%	33.7%	34.7%	22.6%	27.7%	2.90	.72	
Full RTW T2, compared with partial and no RTW, *n* (%)	54 (50.9%)	48.0%	93.1%	62.3%	13.1%	77.3%	28.3%	14.26	.014*	2 > 1, 4, 6; 5, 3 > 4
Partial RTW T3, compared with no and full RTW, *n* (%)	16 (16%)	23.0%	3.3%	12.0%	14.4%	11.1%	29.4%	2.00	.85	–
Full RTW T3, compared with partial and no RTW, *n* (%)	65 (65%)	60.2%	83.5%	73.0%	25.0%	87.7%	41.5%	11.40	.044*	2, 3, 5 > 4

*p < 0.05; **p < 0.01; ***p < 0.001; *M* = mean, *n* = sample size

### Return to Work Status

After 6 months, the proportion of full RTW was significantly higher in the persistently high trajectory (class 2) compared to trajectories with overall lower RTW-SE scores (class 1, 4, and 6; Wald = 14.26, *p* = 0.01). After 12 months, the proportion of full RTW was significantly lower in the decreasing trajectory (class 4) compared with trajectories 2, 3, and 5 (Wald = 11.40, *p* = 0.04). These trajectories were characterized by high baseline scores or quickly increasing RTW-SE levels. This tentatively suggests that higher RTW-SE scores are related to higher RTW rates (Table 3).

### Personal Characteristics

#### Age

Employees in trajectory 1 (low start, moderate increase) and trajectory 5 (moderate start, steep increase) had significantly lower average age compared to employees in all other trajectories (Wald = 46.9, *p* = 0.02). This tentatively indicates that younger employees are associated with trajectories with a higher increase in RTW-SE scores.

#### Gender

Trajectory 2 (persistently high) had significantly more male employees compared to trajectory 1 (low start, moderate increase) and trajectory 6 (persistently low; Wald = 19.35, *p* = 0.002). In addition, trajectory 4 (moderate start, decrease) had significantly more male employees compared to trajectory 1 (low start, moderate increase), trajectory 3 (moderate start, small increase) and trajectory 6 (persistently low). Therefore, being male is associated with persistently high RTW-SE levels and with decreasing RTW-SE levels.

#### Type of MHP

Employees in trajectory 2 (persistently high) had a significantly lower percentage of major depression (Wald = 12.96, *p* = 0.02) and a significantly higher percentage of stress-related disorders (Wald = 114.37, *p* < 0.001) compared to employees in all other classes. In trajectory 4 (moderate start, decrease) employees had a significantly higher percentage of anxiety disorders compared to employees in all other trajectories (Wald = 27.16, *p* < 0.001) (Tables 4, 5).

## Discussion

### Summary

We identified six distinct RTW-SE trajectories in employees with MHPs in the year following sickness leave. Three trajectories were characterized by an increase of RTW-SE levels over time, namely low start, moderate increase (class 1), moderate start, small increase (class 3) and moderate start, steep increase (class 5). The other trajectories were defined by persistently high (class 2), persistently low (class 6) and decreasing scores over time (class 4). Anxiety disorder was more prevalent in the decreasing trajectory, while stress-related disorders and fewer major depression were characteristic for the persistently high trajectory. Furthermore, younger employees were found in the two trajectories showing the highest increase of RTW-SE levels. Moreover, the proportion of employees that had fully returned to work at 12 months, was significantly lower in the decreasing trajectory. Additionally, the trajectories did not differ in their composition regarding education, burnout scores, and work characteristics such as psychological job demands and social support at work.

### Individual Variation in RTW-SE

The identified trajectories differed on RTW-SE baseline levels and their trajectories over the course of 12 months. Around 78% of the employees reached RTW-SE scores of 4.6 or higher after one year (class 1, 2, 3, 5). About 22% of employees attained average scores ranging from 2.6 to 3.2 (class 4, 6), and in about 15% of employees RTW-SE scores decreased (class 4).

Our study identified that the 3 classes that increased over time, did so regardless of their baseline RTW-SE scores (2.2, 2.8 and 3.9) and attained high RTW-SE scores after 12 months (4.6, 5.5, and 4.6). Additionally, we identified classes that showed rather stable scores, both high and low over time (class 2, 6), classes with a steep increasing slope (class 5) and a group with decreasing values (class 4). Lagerveld and colleagues [16] have reported that in general those with lower baseline self-efficacy levels appear to follow a delayed self-efficacy growth curve with lower values at each time point. While this finding possibly reflects the general trend in a sample, the findings in this study add valuable information to this finding with the statistical technique used by showing that RTW-SE scores not necessarily increase over time for all patients. In addition, we revealed that slopes are variable, highlighting considerable individual variability of RTW-SE trajectories and potential risk groups.

### Profiling the Trajectories

The observed differences between employees in the trajectories on work and personal characteristics, and RTW status provide some insight about what different employees may need in terms of RTW support. Although we have found significant statistical differences between trajectories for several variables, our results suggest that particularly type of MHP is an important factor.

Our findings indicate that RTW-SE scores are associated with RTW rates. At 12 months, the proportion of employees that had fully returned to work, was significantly lower in the decreasing trajectory compared to trajectories with high baseline or quickly increasing RTW-scores. This finding is in line with earlier studies reporting that both baseline RTW-SE and RTW-SE increase before full RTW were predictive of a shorter duration until full RTW [16, 17]. Furthermore, RTW rates in the decreasing trajectory declined over time. As relapse is common during the RTW process in about 20% of employees [19], RTW-SE scores may be an important indicator for both relapse and RTW. A possible explanation that no differences were found between RTW-SE trajectories for partial RTW, is that we were not able to measure the nuances of partial RTW (1–99%). Therefore, the difference between someone who returned to work for 20% or 70% for instance, were not detected, which may explain the lack of this finding.

Employees in the decreasing trajectory reported significantly more anxiety disorders than employees in all other trajectories. Previous research also showed some evidence for a relationship between symptoms of anxiety disorders and low self-efficacy [40, 41]. In this group we see that RTW-SE levels decrease between 6 and 12 months after sick leave and RTW rates were significantly lower compared to most other groups. The decrease between 6 and 12 months might be related to employees (partly) resuming their work and being increasingly exposed to stressors in the workplace. If effective coping mechanisms to manage anxiety are not acquired, this could be the time when anxiety symptoms increase and (RTW) self-efficacy subsides. Therefore, it may be valuable to offer treatment that focuses more and earlier on work-related aspects of RTW, such as work-focused cognitive behavioral therapy (W-CBT). Previous research has demonstrated that W-CBT, compared to regular CBT, accelerates functional recovery and time to RTW [18]. W-CBT addresses work issues in an early phase and uses work as a context to reach treatment goals (e.g. activation, time structure, social contact and increasing self-esteem). Additionally, well-guided early RTW and gradual RTW are part of this approach as it enables people to attain the necessary coping skills to deal with (return to) work stressors and practice these skills at work.

Furthermore, the proportion of employees with major depression was significantly smaller in the persistently high trajectory compared to all other trajectories. This finding is in line with prior research reporting that higher levels of depressive symptoms are associated with lower levels of self-efficacy [13, 40, 42]. Therefore, employees with major depression may likewise benefit from interventions that focus on work-related issues in an early phase, such as W-CBT, to stimulate early and gradual RTW, which in turn, if well guided by a professional, can increase the RTW-SE of an employee.

Furthermore, employees in the persistently high trajectory had significantly more stress-related complaints compared to employees in all other trajectories. Stress-related complaints are less severe disorders and may be related more strongly to work characteristics [43]. Moreover, people with stress-complaints may be more confident that they can resume work, if changes in the work environment are made [43]. Depression and anxiety are highly prevalent and relapse-prone conditions, which generally lasts longer [44]. Therefore, employees with these MHPs probably need more extensive treatment to overcome their problems. In addition, models on job demands have indicated that chronic (work) stress can in the long run result in more severe MHPs such as depression and burn-out [45]. Therefore, timely interventions for employees with stress-related complaints may help prevent more severe MHPs in the long run. For instance, it may be important to continue coaching even after employees have returned to work. To strengthen work-related self-efficacy, by practicing effective coping skills in the workplace, may benefit sustainable RTW in the long term [18, 46, 47].

The proportion of men was higher in the persistently high trajectory, but also in the decreasing trajectory. Earlier research has been inconclusive about the relation between gender and work resumption [7, 8]. Furthermore, trajectories that showed the highest increase in RTW-SE had significantly lower average age compared to employees in all other trajectories. This is in line with earlier research [7, 8, 19]. It is important to note however that age, gender and type of MHP are likely to be related. Therefore, differences between RTW-SE trajectories in terms of MHPs may partly be explained by differences in age and/or gender.

Unexpectedly, no difference between trajectories was found for work-related variables such as psychological job demands, decision control, job insecurity, and social support. This finding suggests that work characteristics at baseline may be less important for RTW-SE at a later point. Previous research has indicated that the experience at the workplace and with the employer during the RTW process (e.g. making adaptations to the work, communication with and support from supervisors and co-workers) is highly important for facilitating successful RTW [46, 48].

On a general note, the results of the present study are in line with earlier research in the field. A recent study by Spronken and colleagues on gradual RTW trajectories similarly revealed that more advantageous trajectories are associated with less severe MHPs, lower age and male gender [19]. In practice, focus groups may be valuable to evaluate recognizability of the trajectory groups by employees, employers, occupational physicians, and health care workers. In addition, the trajectory groups can be valuable to advance the knowledge about barriers, expectations and variability that exists during the RTW process [19, 46]. This in turn can increase awareness and foster better communication and social support between all stakeholders.

### Strengths, Limitations and Future Research

A study strength is the longitudinal design, which enabled us to examine the course of RTW-SE in the year following sick leave and produced detailed classes. A large amount of information would have been lost if only one average trajectory was examined. This would have suggested that all employees presented an increase in RTW-SE levels over time. Another strength is the wide range of possible predictors that was tested, which made extensive profiling of the trajectories possible.

A limitation of the present study is that we did not know the exact timing of patients’ RTW, since information about RTW was only assessed at the time points when the questionnaires were sent out. Therefore, the measurement of RTW-SE and RTW might not have taken place at the same moment in time as patients could have returned to work at any time point before the point of measurement. Furthermore, the baseline questionnaire may have been collected later than the actual first sick leave of the employees, as patients were invited to participate in this study during the first meeting with the OP. This limits the range of interpretation that can be carried out, e.g. whether increased RTW-SE levels are preceding RTW. Future studies may benefit from more meticulous measurement about the timing of RTW to advance the understanding about first RTW, relapse, and sustainable work resumption. Nonetheless, knowing the average RTW rates per cluster at each time point still provides invaluable information about the global differences between clusters.

Another limitation of this study poses the relatively small sample size in total and particularly of the two smallest trajectories. However, our sample shows to be representative of the Dutch population regarding age, education and type of MHP [39]. Various working sectors are represented, yet men are slightly underrepresented. Patients had multiple ratings for MHPs, which made exact comparison with the average population infeasible. However, as comorbidity with other mental health problems is highly prevalent in the average population (46.3%), overall our sample represents the Dutch population well. Nonetheless, the results need to be interpreted with caution because the small sample size may have led to a diminished power to detect differences between the trajectories. However, small classes might not be avoidable completely in latent group-based modeling, if a comprehensive understanding of the data is the goal [49]. Thus, additional research with bigger samples is needed to confirm the obtained classes.

Moreover, we calculated a continuous score for the burnout scale (UBOS), which might have led to a loss of information compared to using the emotional exhaustion scale alone. However, Cronbach’s alpha was high in the present study, demonstrating good internal consistency.

Although our data included many variables and were a rich source of information on RTW-SE courses, the data were gathered in the context of another study. Therefore, information that would be valuable in this study was not necessarily included. For instance, there was no information on motivational aspects and treatment employees received during their RTW. Hence, future studies may examine whether trajectories differ on additional psychological and human work-related characteristics such as treatment, work engagement, social support, and job crafting [46, 48]. In addition, future research that examines the association between RTW-SE trajectories and gradual RTW arrangements may be very valuable [19].

Furthermore, the follow-up period of 12 months does not allow us to evaluate the long-term course of RTW-SE levels and related variables, such as sustainable work resumption. Additionally, pre-sick leave measurements are not included, which could be valuable since we know that self-efficacy possesses trait-like and situation specific properties [41]. Therefore, future multi-wave studies are needed that include a pre-sick leave measurement and longer follow-up periods to allow for a more encompassing exploration of RTW-SE trajectories and related variables.

## Conclusion

In conclusion, we found significant variability in RTW-SE trajectories among employees with MHPs. Our results indicate that stress-related complaints and less severe MHPs are related to advantageous trajectories. Therefore, timely intervention may prevent the development of more severe complaints. Furthermore, employees with anxiety disorder and major depression have overall lower RTW rates and may especially benefit from extensive and ongoing interventions, particularly once returned to work. Furthermore, our study supports a strong link between RTW-SE and actual RTW. The insights into different RTW-SE trajectories and their characteristics contribute to the improvement of personalized RTW treatment for employees with MHPs.

## Data Availability

The data that support the findings of this study are available from the corresponding author [LH] upon reasonable request.
